# Point of care susceptibility testing in primary care - does it lead to a more appropriate prescription of antibiotics in patients with uncomplicated urinary tract infections? Protocol for a randomized controlled trial

**DOI:** 10.1186/s12875-015-0322-x

**Published:** 2015-08-21

**Authors:** Anne Holm, Gloria Cordoba, Tina Møller Sørensen, Lisbeth Rem Jessen, Volkert Siersma, Lars Bjerrum

**Affiliations:** Department of Public Health, The Research Unit for General Practice and Section of General Practice, University of Copenhagen, Øster Farimagsgade 5 opg. Q, PO box 2099, 1014 Copenhagen K, Denmark; Department of Veterinary Clinical and Animal Sciences, Faculty of Health and Medical Sciences, University of Copenhagen, Dyrlægevej 16, 1870 Frederiksberg C, Denmark

## Abstract

**Background:**

Urinary tract infection (UTI) is a common infection in primary care and is the second leading reason for prescription of antibiotics in Denmark. The diagnosis is often based on symptoms and urine dip-stick, which has limited validity, causing the risk of unnecessary antibiotic prescription. Additionally, with increasing antibiotic resistance, the risk of choosing an antibiotic to which an infecting pathogen is resistant is rising. Combined point-of-care-tests (POCT) for urine culture and susceptibility testing have been developed and validated for primary care, and performing such a test in all patients with suspected UTI in primary care seems rational in order to reduce the use of inappropriate antibiotics. However, the clinical effect of the culture and susceptibility test has not yet been investigated. This study aims to investigate whether POCT urine culture and susceptibility testing decreases the inappropriate use of antibiotics and leads to faster patient recovery.

**Methods/design:**

Randomized controlled open label trial of two diagnostic approaches. 750 patients with symptoms of uncomplicated UTI, consecutively contacting their general practitioner (GP), randomized to either POCT urine culture and susceptibility testing and targeted treatment or POCT urine culture without susceptibility testing and empirical treatment. Treatment is started when the POCT is read. The two groups are compared with regard to appropriate choice of antibiotics, clinical remission, and microbiological cure rates.

**Discussion:**

The results of this study may provide important evidence to recommend POCT culture and susceptibility testing in all patients with suspected uncomplicated UTI. This could become an additional strategy to fight antibiotic resistance.

**Trial registration:**

ClinicalTrials.gov NCT02323087.

## Background

Antibiotic resistance is rapidly spreading, making it one of the most serious threats to human health. The World Health Organization has stated that a post-antibiotic era is a very real possibility and that urgent actions are needed in order to maintain the effect of antibiotics [1].

Primary health care in Denmark is responsible for about 90 % of all redeemed prescriptions of antibiotics, and it is known that a high out-patient consumption of antibiotics leads to high levels of resistance [2, 3]. Thus, a cornerstone in the efforts to reduce antibiotic resistance is to reduce and improve prescription of antibiotics in primary health care.

In 2008, 1.8 % of all patients consulting their GP in Denmark were diagnosed with a UTI [4]. Resistant strains of *E. Coli*, which is the causative organism in 70–80 % of all UTIs, are spreading world-wide [5, 6]. In Denmark, 33–40 % of *E. Coli* isolated from urine samples from primary care are resistant to sulfamethizole and 6–10 % to pivmecillinam, which account for about 80 % of all antibiotic treatments of adults with UTI in primary care in Denmark [3, 7]. It is, therefore, critical that a UTI is treated only when clinically indicated and using an appropriate antibiotic, i.e., one to which the infecting pathogen is susceptible, taking into account the use of first-line agents over second-line agents.

Urine culture is necessary to accurately determine if a patient has a UTI since other tests have limited predictive values in primary care and treating based on symptoms can cause up to 50 % being inappropriately treated [8, 9]. Susceptibility testing adds the advantage to predict whether a first-line antibiotic can be expected to eliminate the infecting pathogen. However, delaying treatment for several days while waiting for the results of the susceptibility test cannot be justified as symptoms are painful and affect quality of life [10, 11]. Point of care test (POCT) culture and susceptibility testing provides the result within 24 h, and can, therefore, be used to target individual therapy without compromising patient welfare. Inappropriate antibiotic prescribing can be partly avoided by performing a POCT culture since this will assumedly eliminate treatment of patients without bacteriuria. However taking into account the above-mentioned resistance rates in *E. Coli* and for example enterococci being inherently resistant to both antibiotics, this could result in about 20–30 % inappropriate antibiotic prescriptions for UTI. Adding susceptibility testing to the POCT should raise the appropriate antibiotic prescriptions above 90 %.

This study aims to answer the questions: 1) Does POCT urine culture and susceptibility testing decrease the use of inappropriate antibiotics, and 2) Does targeted therapy improve clinical outcomes in patients with suspected uncomplicated UTI in general practice when compared to POCT urine culture without susceptibility testing? We hypothesize that the use of POCT susceptibility testing improves the following outcomes: Appropriate choice of antibiotic, clinical remission, and microbiological cure rate.

## Methods

### Study design

Randomized controlled open label trial of two diagnostic approaches in a primary care setting.

### Recruitment process

General practitioners (GPs) 200 general practices in the Copenhagen area will be contacted by letter with the aim of recruiting 50 GPs. All GPs will receive relevant training in the use of POCT culture and susceptibility testing, and their skills will be validated using an online test on how to read the POCT.

#### Patients

Patients presenting with symptoms of UTI will be recruited at the general practice during consultation. To ensure interpretation of POCT within 24 h, only patients contacting practice from Monday to Thursday will be included. Each GP will recruit and randomize 15 patients.

#### Inclusion criteria

Female adult patients, 18 years or older, presenting at their GP with dysuria, frequency or urgency, which has been present for 7 days or less, and for which the GP suspects uncomplicated UTI (including recurrent UTI, uncomplicated diabetes mellitus defined as orally treated, well regulated and without secondary complications, and elderly patients). Patients should be able to deliver a mid-stream urine sample, to provide informed consent, and should be willing and able to fill out a symptom diary.

#### Exclusion criteria

- Negative dip-stick analysis on leucocytes and nitrites (to reduce the number of negative cultures)
- Complicated UTI
  - Known pregnancy
  - Severe systemic symptoms, high fever, flank pain
  - Recent bladder surgery (within past 4 weeks)
  - Urinary tract abnormalities
- Serious systemic disease
  - Life-threatening cancer
  - Insulin-dependent diabetes
  - Long-term corticosteroid treatment
  - Other conditions with compromised immunity
- Former participation in the study
- Patients presenting on a Friday (since POCT is read after 24 h)

#### Randomization and groups

The patients are block randomized in blocks of 10 to ensure approximately equal sizing of the groups. The randomization group for each patient is placed in a sealed envelope which is opened either during or after consultation.

- For the intervention group, POCT culture and susceptibility testing is performed. Treatment is based on the result of the susceptibility test and clinical guidelines.
- For the control group, POCT culture without susceptibility testing is performed, and treatment is based on clinical guidelines.

### Informed consent

All patients receive oral and written information before signing informed consent forms.

#### Screening logs

All participating general practitioners, secretaries, and nurses will be asked to maintain an anonymous screening log of all patients fulfilling the inclusion criteria in the inclusion period. This will be used to assess selection and its effect on the study results and for the attrition flow chart.

### Data collection

#### Case-report form

After oral and written information about the project and written consent to enrollment, the GP will take a structured history and fill out a case report form. Data from day 1 consist of:

- Name and social-security number
- Drug allergies
- Diabetes
- Number of UTIs within past year
- Symptoms of UTI
  - Dysuria
  - Frequency
  - Urgency
- Duration of symptoms
- Randomization group

The patients are asked to contact the GP the next morning by telephone or e-mail for treatment. The patients are also asked to contact the GP if symptoms persist after 4–5 days. The GP can advise on painkillers if necessary.

The next day, the GP will read the plate and inform the patient about the result and potential treatment with antibiotics. The GP will complete the case report form including the following data:

- Reading of culture plate
  - No significant growth of uro-pathogens
  - Significant growth of at least one uro-pathogen
  - Inconclusive
- For identified uro-pathogen(s):
  - Species
  - Amount in cfu/mL
  - Resistance pattern towards trimetroprim, sulfamethizol, ampicillin, nitrofurantoin, and pivmecillinam (intervention group)
- Treatment:
  - Name, dose, and duration of antibiotic

#### Symptom diary

The patients are asked to compile and return a paper symptom diary. Through personalized text messages, they are reminded on day 3 to fill out the diary and on day 7 to send it to the Section of General Practice, University of Copenhagen. If they have not sent the diary on day 10 or do not have a cell phone, they receive a phone call. The diary has been face- and content validated through focus groups and personal interviews. The scales for symptom severity, bothersomeness and impact on daily activities are currently under psychometrical validation using the partial credit Rasch model for polytomous items. The secondary outcome of clinical cure is measured using a single item where the patient by the end of each day answers if her symptoms of urinary tract infection are completely gone. The scales for symptom burden are not a part of the secondary outcome but serves to improve the patient’s evaluation of her own cure.

The diary measures:

- Employment status, job and number of employees
- Use of medication other than antibiotics and painkillers
- Symptom severity on day 1–7
- Symptom bothersomeness on day 1
- Impact on daily activities on day 1
- Use and possible change of antibiotics on day 1–7
- Use of painkillers on day 1–7
- Re-consultation with their GP/out-of-hour service on day 1–7
- Sick-leave on day 1–7
- Day of becoming symptom-free

#### Urine samples

A mid-stream urine sample from the day of consultation will be divided in two. One part is sent to the local microbiological department, and the other part will be examined at the general practice using the POCT. On day 14 another urine sample will be sent to the local microbiological laboratory for culture.

#### Microbiological analyses performed at the microbiological laboratory– Gold standard

A mid-stream urine sample from day 0 to day 14 are analyzed at the local microbiological laboratory. The sample from day 0 serves as a quality control of the culture and susceptibility testing performed in general practice. The sample from day 14 is the microbiological outcome measure. The samples are transported to the microbiological laboratory in Urine-Monovette® (Sarstedt) containing boric acid to stabilize the bacterial count.

At the microbiological laboratory urine sample are dispersed on Inoqul A™ Bi-plate (CHROMagar and blood agar) with 10 μL on each half of the agar. The susceptibility pattern is determined on Mueller Hinton agars with disks containing mecillinam, cefpodoxim, cefuroxim, gentamicin, piperacillin + tazobactam, meropenem, ampicillin, nalidixic acid, trimethoprim, nitrofurantoin, sulfamethizol, and vancomycin. All samples are quantified. If the bacterial count on the two agars on Inoqul A™ differs with more than a factor 10, the procedure is repeated.

Significant growth is defined as growth of ≥10^3^ colony forming units per millilitre (cfu/mL) for *E. coli* and *S. saprofyticus*, ≥10^4^ cfu/mL for other typical uro-pathogens and ≥10^5^ cfu/ml for possible uro-pathogens following current consensus [12]. All pathogens with significant growth are identified and susceptibility pattern determined. Any pathogen growing at least 10^3^ cfu/ml, unless the above mentioned criteria are fulfilled, is classified as contamination, and in these cases the susceptibility pattern is not determined. Insignificant growth is defined as ≤ 10^2^ cfu/mL or less. Susceptibility pattern is determined according to EUCAST and NordicAST recommendations. The internal quality control is performed measuring inhibition zones on chosen reference strains from American Type Culture Collection (ATCC) and National Collection of Type Cultures (NCTC).

#### Microbiological analyses performed on-site at the general practice

##### Culture (control group)

Point-of-care culture will be performed using ID Flexicult™ (SSI DIagnostica, Denmark) which is a chromogenic agar plate for identification and quantitation of urinary tract pathogens. The sample is seeded with a 10 μL inoculation needle, the lid is applied, and the agar plate incubated upside down at 35 °C overnight. The plate is read the next day. If it is positive, no further incubation is needed, if it is negative, incubation is continued until 24 h after inoculation. The bacterial identification is based on colony color and size. The agar plate can be seen on the right side of Fig. 2.

##### Culture and susceptibility testing (intervention group)

Urine culture and susceptibility testing will be performed on the intervention group by means of a POCT, the FLEXICULT™ SSI-Urinary Kit (SSI Diagnostica, Denmark). The kit is a chromogen agar in an ordinary Petri dish, but with higher sides. The Petri dish is divided into 6 compartments: 1 large compartment for quantitative analysis and 5 smaller compartments for susceptibility testing. The agar in each of the smaller compartments contains 1 of 5 antimicrobials: trimethoprim, sulfamethoxazole, ampicillin, nitrofurantoin, and mecillinam. The agar plate can be seen on the left side of Fig. 2.

The agar plate is flooded with the urine specimen for a couple of seconds and then incubated at 35 °C over night. The plate is read on the following day.

As the concentrations of the antimicrobials in the 5 smaller compartments are adjusted in accordance with breakpoints, growth on these compartments indicates resistance of the pathogen in question and hence a potential risk of treatment failure.

**Fig. 1:**
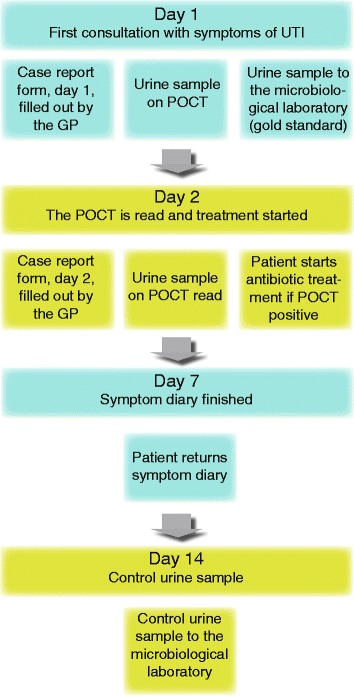
Flow-chart for data collection. POCT: Point of care test. This refers both to POCT culture and POCT culture and susceptibility testing. GP: General practitioner

**Fig. 2:**
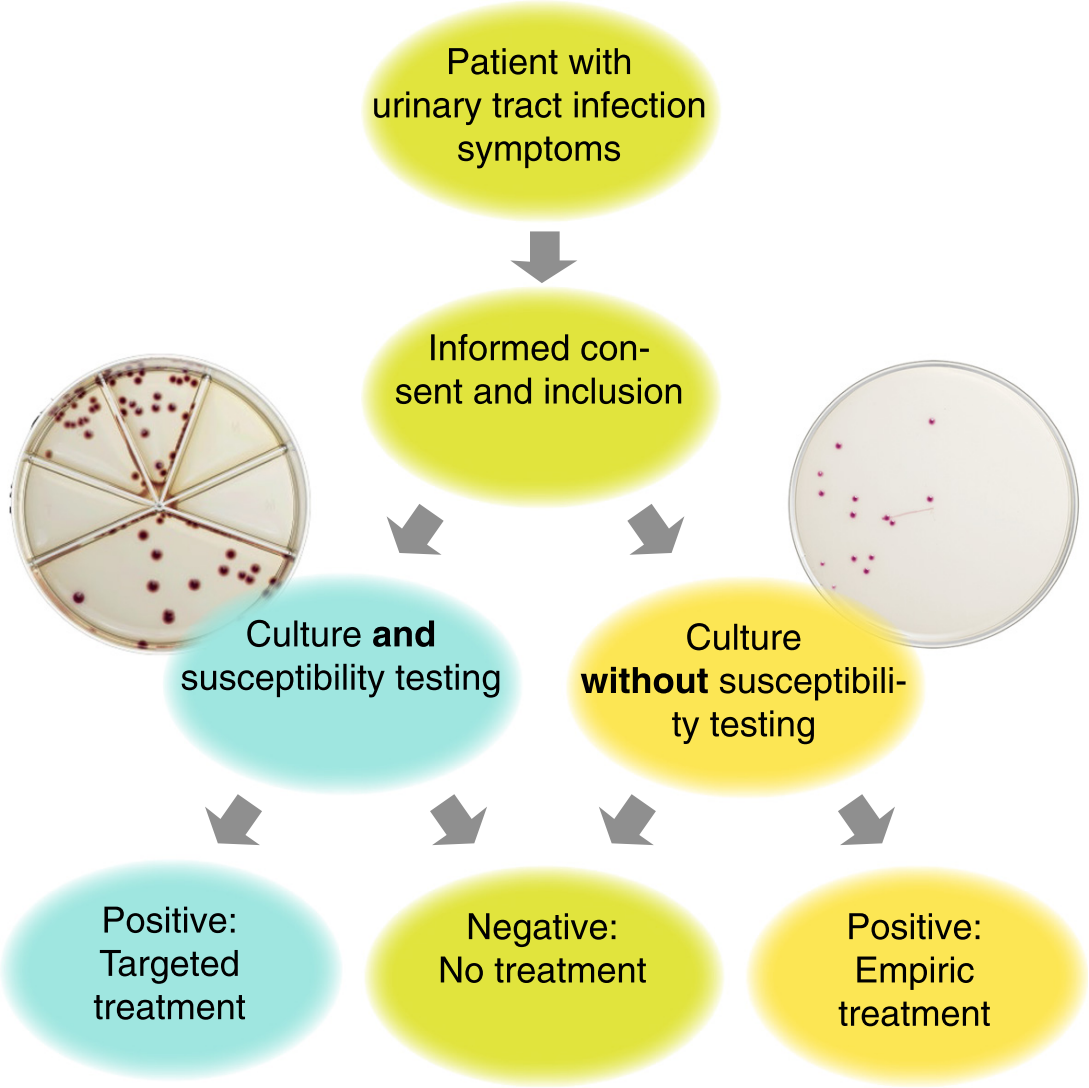
Study design of randomized controlled trial. The figure shows the study design with the intervention arm on the left and the control arm on the right. The shown agars are the ones used in the trial, courtesy of SSI Diagnostica

Figure 1 illustrates the data collection process and Fig. 2 explains the study design and the difference between the intervention and control arm.

### Definition of outcomes

#### Primary outcome

- The proportion of patients receiving appropriate antibiotic treatment on the day after consultation. Data obtained from case-report form.

**Fig. 3:**
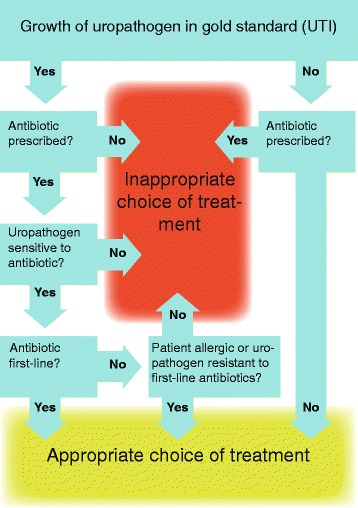
Short title of figure: Flow-chart for primary outcome and definition of appropriate and inappropriate choice of treatment

Appropriate antibiotic treatment is defined as receiving a first-line antibiotic to which the infecting organism is susceptible, if there is significant growth in the gold standard or receiving any antibiotic to which the infecting organism is susceptible if there is significant growth in the gold standard if the patient is allergic or the infecting organism is resistant to all first-line antibiotics or not receiving an antibiotic if there is no significant growth in the gold standard. The definition is illustrated in Fig. 3.

#### Secondary outcomes

- The proportion of patients who are asymptomatic on the fourth day of treatment (clinical cure) defined as the patient stating, her symptoms are completely gone regardless of symptom score. Data obtained from symptom diaries
- The proportion of patients with no significant bacteriuria on day 14 (bacteriological cure). Data obtained from control urine sample

### Ethical aspects and patient safety

The study has been approved by the Ethical Committees for the Capital Region of Denmark and reported to the Danish Data Protection Agency. All patients entering this study receive a higher level of diagnostics and treatment than standard care at the moment. The improved diagnostics and, thereby, the reduction of overtreatment will benefit the individual patient more than the disadvantage of delaying treatment. All data are kept under the same security as other sensitive data at a GP office. In case of any adverse event that could be attributed to participation in the trial (eg. worsening of symptoms due to delay of treatment), the GP in charge of care of the participant will follow a flow-chart to determine if the trial-responsible investigator should be notified and how fast. If the event is considered harmless or unlikely to be related to the trial, it is registered on the case-report form. If it is considered serious and likely related to the trial, the trial coordinator is contacted by telephone within 24 h. At least two members of the trial team evaluate serious events related to the trial and decide if the trial team should be gathered. All adverse events that could be attributed to participation in the trial are recorded and analyzed biannually by the coordination team. All results, positive, negative, and inconclusive, will be published.

### Analysis

#### Sample size calculation

##### Primary outcome

The proportion of appropriately treated patients in the control group is assumed to be 70–80 % based on an assumption that POCT culture will be precise in determining UTI, but current local resistance rates in *E. Coli* (70–80 % of infections) of about 6–10 % to pivmecillinam (50 % of patients with UTI) and 30–40 % to sulfamethizole (30 % of patients with UTI) will result in inappropriate treatments as defined in Fig. 3 [3, 7]. To detect a statistically significant (*α* = 0.05) 10 percentage-point difference between the two groups with 80 % probability, assuming an intra-class correlation of 0.2 between patients in the same practice, a sample of 600 patients is needed. In order to take possible drop-outs and sub-analyses into account, the study aims to enroll 750 patients.

### Secondary outcomes

#### Clinical remission

McNulty and Ferry reported clinical cure rates of 69 % on day 5 after targeted treatment with trimethoprim and 44 % on day 5 empiric treatment with pivmecillinam respectively in patients with uncomplicated UTI [13, 14]. Assuming a cure rate of 60–70 % on the fourth day of treatment (day 5) in the intervention group, a difference of at least 15 percentage points could be detected with the chosen sample while accounting for a 25 % drop-out on clinical follow-up.

#### Bacteriological cure rate

Since bacteriological cure with empiric antibiotics on day 8–10 is about 90 % [14] as reported in a Swedish study, we are not expecting to see a significant difference between the groups regarding this outcome.

### Statistical analysis

Comparison of the two randomization groups for both the primary and the secondary outcomes will be done by means of an odds ratio (OR) from a logistic regression model; clustering within practices is adjusted for by generalized estimating equations (GEE). Analyses will be performed intention-to-treat, i.e., the patients are analyzed in the groups they are randomized to regardless of the treatment they actually received. Effect modification – whether the effect of the intervention differs between subgroups in the data – will be investigated for GP factors (organization of practice, performance in reading the POCT), patient factors (age, concurrent illness, socio-demographic data, initial symptom score) and microbiological factors (amount and species). If a sufficient sample is obtained, sub-group analysis will be performed for patients with diabetes, elderly patients and patients with recurrent UTI since these groups are expected to benefit the most from the intervention. In an additional analysis of the primary outcome, the group inappropriately treated will be divided into under-treated and over-treated and analyzed in multinomial logistic regression models. Comparison of cure-rates will be done with Kaplan-Meier curves and log-rank tests. A P-value of 0.05 will be considered significant. Analyses will be performed with SAS v9.4.

## Discussion

In Denmark, POCT combined culture and susceptibility testing has been in use for decades, and the use has increased since introduction of the FLEXICULT™ SSI-Urinary Kit. Despite this popularity no clinical trials have yet validated its impact in clinical practice. This study will investigate the effect of POCT susceptibility testing on appropriate choice of antibiotics and on clinical and microbiological cure in patients with uncomplicated UTI in primary care in Denmark.

The clinical effect and cost-effectiveness of POCT culture and susceptibility testing in UTI is currently being investigated by another research group [15]. Although both studies aim at investigating the effect of the Flexicult on the appropriateness of antibiotic use and the impact on patient outcomes, there are at least three important differences. Firstly, in the study by Bates et al., the effect of combined culture and susceptibility testing is compared to various forms of standard care in a four-country multicenter setting. The focus of the present study is narrower, specifically aiming at determining the value of the susceptibility component compared to culture alone in a single region in Denmark. Secondly, all GPs in this study are experienced users of POCT susceptibility testing, and their skills are validated before enrollment of patients as described under the recruitment process, thus inter-practice variation is minimized. Thirdly, in this study, both groups will have treatment delayed until a positive culture is obtained, thereby minimizing the number of culture-negative patients receiving inappropriate antibiotic treatment.

We have chosen to include patients with diabetes, recurrent UTI and elderly patients when they are otherwise healthy and can be safely included. In the analysis, they are investigated for effect modification and, if the sample allows it, they are analyzed separately, since they could be expected to benefit more from the intervention than other groups.

A challenge of this study is the similarity between the intervention and control groups. The potential difference between the groups in this study will mainly be driven by those patients in the control group receiving an antibiotic to which, the infecting pathogen is resistant. Since *in vitro* resistance rates in Denmark against the most commonly used antibiotics for UTI are 15–40 %, the effect could turn out minor at present [3]. If the study detects no additional benefit of susceptibility testing over culture alone, this will provide important information for the Danish national health care system. However, the results may not be directly applicable to countries outside Scandinavia. On the other hand, if susceptibility testing proves superior to culture alone, the impact of such a finding will likely be much higher in countries where resistance rates are higher. In conclusion, the present study will test the hypothesis that POCT susceptibility testing for uncomplicated UTI and individually targeted therapy will decrease the use of inappropriate antibiotics and positively influence clinical cure rates. If this proves true, the results of the study may provide important evidence to recommend POCT susceptibility testing for patients with suspected UTI. This could become one of many strategies to fight antibiotic resistance.
